# A review on recent advances in hydrogen energy, fuel cell, biofuel and fuel refining via ultrasound process intensification

**DOI:** 10.1016/j.ultsonch.2021.105536

**Published:** 2021-03-22

**Authors:** Ujwal Kishor Zore, Sripadh Guptha Yedire, Narasimha Pandi, Sivakumar Manickam, Shirish H. Sonawane

**Affiliations:** a Department of Chemical Engineering, National Institute of Technology, Warangal, Telangana 506004, India; b Petroleum and Chemical Engineering, Faculty of Engineering, Universiti Teknologi Brunei, Bandar Seri Begawan BE1410, Brunei Darussalam

**Keywords:** Ultrasound, Hydrogen, Production, Storage, Fuel cell, Biofuel

## Abstract

Hydrogen energy is one of the most suitable green substitutes for harmful fossil fuels and has been investigated widely. This review extensively compiles and compares various methodologies used in the production, storage and usage of hydrogen. Sonochemistry is an emerging synthesis process and intensification technique adapted for the synthesis of novel materials. It manifests acoustic cavitation phenomena caused by ultrasound where higher rates of reactions occur locally. The review discusses the effectiveness of sonochemical routes in developing fuel cell catalysts, fuel refining, biofuel production, chemical processes for hydrogen production and the physical, chemical and electrochemical hydrogen storage techniques. The operational parameters and environmental conditions used during ultrasonication also influence the production rates, which have been elucidated in detail. Hence, this review's major focus addresses sonochemical methods that can contribute to the technical challenges involved in hydrogen usage for energy.

## Highlights

• A detailed review of ultrasound enabled mechanism and its disparate applications have been presented.
• Emphasis is made on the sonochemical routes for hydrogen production and their advantages.
• Recent research studies on the synthesis of materials for hydrogen storage have been reported.
• Sonochemistry relevance in the fuel cell, biofuel technology and fuel refining have been discussed.

## Nomenclature

E_1/2_: Half-wave potential
ECSA: Electrochemical surface area
OCP: Open circuit potential
SA: Surface area
MA: Mass activity
REP: Reformer electrolyser purifier
SOEC: Solid oxide electrolysis cell
DBPS: Dual bed photocatalysis system
PEM: Proton exchange membrane electrolysis
BET: Brunauer–Emmett–Teller

## 1 Introduction

Sonochemistry is an emerging research discipline that focuses on studying ultrasound effects for its physical and chemical transformations. Ultrasound typically in the range of 20–10^4^ kHz, is used in the sonochemical synthesis. The chemical aftermath produced as a consequence of ultrasound is major due to cavitational effects. The detailed theory on cavitation has been extensively reported [1], [2]. The acoustic cavitation involves three stages of nucleation, bubble growth and implosion. This leads to two effects: a chemical effect which includes the generation of radicals, while the physical effect includes agitation, turbulence, mass transfer, microstreaming, shockwaves, etc.

Formation of cavities occurs in the liquid because of the interaction of pressure waves (ultrasound) with the liquid medium. These cavities undergo continuous compression and rarefaction when they interact with positive and negative pressure cycles. This continues until the cavities reach a critical radius, which depends on the frequency of ultrasound. Once the cavities reach this radius, they no longer absorb energy from the sound field. As a result, it cannot remain stable, and the surrounding media comes in, and the bubbles implode. The implosion of cavities provides a unique chemical reaction environment at high temperatures, high pressures, and cooling rates [1]. The experimental results demonstrate that bubbles' implosion creates a local temperature of 5000 K and pressures as high as 1000 atm [1]. Further, the simulation results were reported by Nilesh et al. [3] for acoustic cavitation, while Senthil Kumar and Pandit [4] reported for hydrodynamic cavitation. For acoustic cavitation, Flynn’s criterion and Rayleigh-Plesset equation for single cavity were utilised. It suggests that the ultrasound effect is optimum at lower intensities (above a threshold value) and higher frequencies. It was also reported that the maximum cavitational efficiency that can be reached is 20%. For hydrodynamic cavitation, the concept of cavity cluster showed better insights than an isolated single cavity. The simulations could suggest optimised operation variables, and the application of high/low inlet pressure, high/low recovered downstream pressure and dissolved gases was also elucidated.

Ultrasound exhibits profound effects on chemical reactions, attributed to the hot spot and electrical theories [5]. The hot spot theory suggests that the enhancement of reaction rates is due to the conformation of local hot spots due to the implosion of cavities. The electrical theory attributes special conditions to the accumulation of charge on the separating bubble [6].

The effects of cavitation on different reacting systems are as follows:

1. Homogeneous reaction systems: The implosion of cavities leads to the generation of shock waves, resulting in the disruption of solvent structure/impurities/water molecules. This causes the formation of highly reactive species such as radicals, carbines, etc. This is attributed to the hemolytic fragmentation of species due to high temperatures, and pressures of shock waves and the media shell are also suggested to be an active site of reaction [1].
2. Heterogeneous reaction systems: The application of ultrasound profoundly impacts the liquid–liquid emulsification reactions. The experiments showed enhanced reaction rates due to increased contact areas. In many phase-transfer reactions, ultrasound is solely used, thus eliminating the use of catalysts [5], and the disruption of liquid films leads to reducing the diffusional resistance.

The liquid–solid system is the most widely researched area in sonochemistry. The localised cavitation on the solid surface results in liquid jet release impacting the surface, creating erosion/pitting. This phenomenon finds its application in ultrasonic cleaning. Pitting also results in the exposure of new reactive spots, thereby leading to increased reaction rates. Suslick reported on the melting of metals such as Cu, Zn, Fe, Cr, Ni, etc., at the collision point [1]. He also showed the removal of oxide coatings from Zn, Ni, and Cu. On similar lines, high-intensity focused ultrasound (HIFU) uses cavitation phenomena to ablate the unwanted tissues/tumours in the body, referred to as ‘histotripsy’. In this, the shock waves arising from implosion results in focused boiling, followed by evaporation of the tissue [7].

Sonochemistry has been applied for the synthesis of composites for energy storage applications. For the electrodes of a fuel cell, composite nanomaterials were synthesised, utilising platinum and ruthenium [8], gold and platinum [9], etc. which exhibited enhanced electrical properties. Further, the ultrasound application for producing a homogeneous mixture of Pt/C catalyst was elucidated by Takashi and Kocha [10]. Similarly, for the lithium-ion battery’s electrode material, Cu_2_O-Graphene-based binary nanocomposite [11], graphene oxide-Fe_2_O_3_ based ternary nanocomposite [12], etc. were synthesised. Dipanwita et al. [13] and Shahram et al. [14] reported on the ultrasound-assisted synthesis of MnO_2_-graphene-based binary composites, and competent cyclic stability, specific capacitance and other fundamental electrical properties required for the preparation of electrode material of an energy storage device were noted. Following the ultrasound-assisted route, high reaction rates were attained, which results in time-efficient synthesis [5]. While utilising less time and energy, it resulted in nanocomposites' production possessing uniform distribution and uniform/adequate sizes, thereby making sonochemistry, energy-efficient and time-efficient compared to methods such as mechanical attrition, electro-deposition, etc. [1], [15].

Similarly, Gagol et al. in their review on the application of cavitation for wastewater treatment described in details how ultrasound waves (acoustic cavitation), as well as pressure waves (hydrodynamic cavitation) in combination with other advanced oxidation processes (AOP’s) for wastewater treatment, have been proved to be advantageous for oxidising the organic pollutants as a result of synergistic effect. Additionally, they demonstrated the effectiveness of various processes in oxidising the organic contaminants, dependencies on the process parameters, and suggesting the propitiousness of combining ultrasound technology with the AOP’s [16].

Because of the cavitation effects (local high temperature, maximal pressure and intense micromixing) [17] during sonochemical processes, the electrochemical, mechanical properties, etc. of the formed products are enhanced. The enhancement in the electrical properties was found in supercapacitors. The electrode material formed from nanocomposites using a sonochemical technique possessed higher power density and energy density, high cyclic stability and high specific capacitance compared to the nanocomposites prepared using conventional methods such as the hydrothermal method. This could be due to abnormal conditions created due to the cavitational effects such as intense macromixing, high temperature and pressure. Further, it has been reported that the synergistic effect caused by the ultrasound-assisted synthesis of binary/ternary nanocomposites resulted in enhanced capacitive stability [18]. Arulmani et al. [19] reported the specific capacitance of Ni_2_/PANI prepared both by sonochemical and conventional methods. They observed that the specific capacitance of binary nanocomposites synthesised using ultrasonic-assisted approach was around 1.6 times greater than that of pure polyaniline (PANI) and about 1.3 times greater than the nanocomposites synthesised using the conventional method. Today, the maximum specific capacitance reached using the supercapacitor electrode material prepared through sonochemical method is ≈1000-1200F/g whereas for the hydrothermal method it is ≈80-100F/g and for the solvothermal method it is ≈200 F/g [20], [21], [22]. An enhancement in the mechanical properties was also observed when the material was prepared using the sonochemical process. In the same way, they possessed high tensile strength, young’s modulus, thermal conductivity and crystallinity [23], [24].

**Fig. 1:**
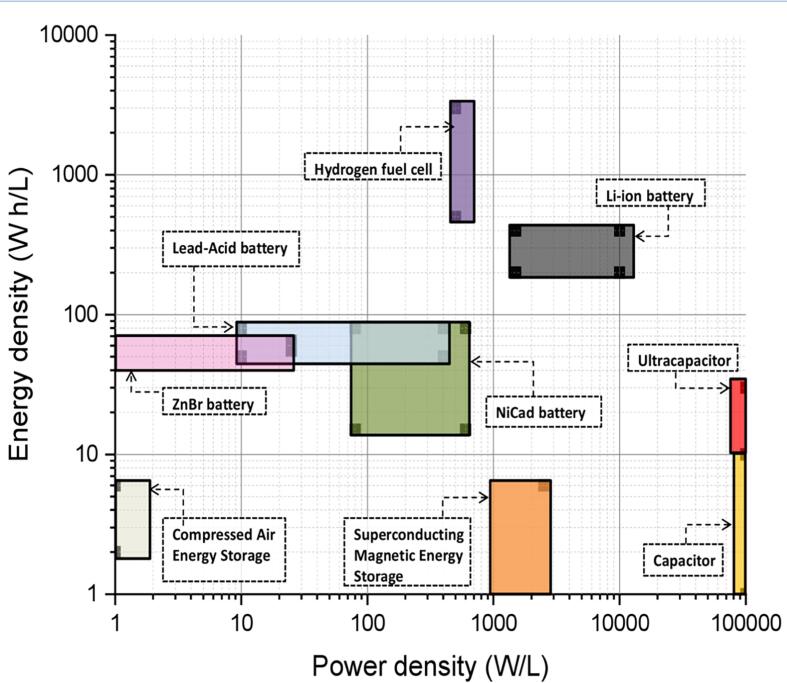
Comparison of energy storage devises based on energy density and power density [26], [27], [29]

Since the beginning of the revolution in the domain of energy storage devices, researchers are attempting to make use of the technologies better by employing cutting edge technology and knowledge while keeping in mind the benignness of the process and the product. Xing et al. (2015) meticulously studied and coherently provided a clear picture of all the modern storage technologies available in the market and suggested the suitability of those technologies for applications [25]. Although further investigations are continuously being conducted and the electrical properties of those technologies are enhanced day-by-day, they are most appropriately and closely associated with a range of values to most of the properties of the energy storage devices. As depicted in Fig. 1, the values of energy density and power density of various state-of-art storage devices have been compared. The compressed air energy storage (CAES), a commercialised electrical energy storage device has been profoundly used for applications requiring good partial-load performance and moderate speed of responses, which showed a power density of 1 W/L and an energy density of 2–6 Wh/L [26]. Superconducting magnetic energy storage (SMES) system has been proven to be advantageous as its discharging property completely for thousands of full-cycle without degrading significantly showed power density of 2500 W/L and energy density of 6 Wh/L [26]. The problems encountered were high cost and environmentally unfriendliness. After this, various battery systems were introduced, and rechargeable were the ones which stormed into the market and gained popularity. Out of the many battery energy storage systems, zinc bromide (ZnBr) flow battery possessed a power density of 25 W/L. An energy density of 55–65 Wh/L, nickel–cadmium (NiCd) based system possessed a power density of 80–600 W/L and an energy density of 15–80 Wh/L lead-acid batteries exhibited a power density of 10–400 W/L and an energy density of 50–80 Wh/L and much profound and used Li-ion batteries showed a power density of 1,500–10,000 W/L and an energy density of 200–400 Wh/L [26], [27].

Further, as the technology became more advanced, researchers devised hydrogen fuel cells capable of stationary or distributed power and transportation power, while being 100% green, thus offering power independence and capacity for energy production. A favourable energy density of 500–3000 Wh/L and a power density of 500 W/L has been demonstrated [27]. Many developed and developing nations are shifting their focus towards these technologies due to its merits. Capacitors are profoundly examined for their huge power density as they possess a short charging period and showed a power density of>1,00,000 W/L and an energy density of 1–10 Wh/L [27]. Supercapacitors exhibit a good blend of the properties of a capacitor and an electrochemical battery that demonstrate a power density of>1,00,000 W/L and an energy density of 10–30 Wh/h, which showed three times better than a conventional capacitor [26]. With the ever-increasing population, the needs are increasing, and lifestyles are getting better, and fulfilling these, industries employ advanced and cutting edge technologies. In the energy domain, industries require reversible storing and rapidly releasing of charge, higher power charge–discharge rate in a short period, etc. which made to replace supercapacitors for conventional batteries to be favorable owing to their numerous desirable qualities. Additionally, being economically available and viable make them the best substitutes [28].

Application of ultrasound has been seen in unique water sensors where electrically conductive polymer composites possess promising properties such as excellent processability and lightweightedness, can permit the execution of a sensor's function by assimilating it into the fabrication process of the structural elements [30]. Additionally, ultrasound finds its industrial application in food processing, the pharmaceutical sector, the medical sector in biomedical devices, ultrasound imaging, etc. Further, sonolysis, which is the formation of reactive species when ultrasound reacts with water, has received its application in water treatment and its purification. Bhargava et al. in their review paper on food processing have expounded how ultrasound, unlike conventional techniques, doesn’t let product quality degrade and offers advantages like better shelf-life while being able to retail the product characteristics, reduced time and high efficiency [31]. Similarly, Gagol et al. in their review paper on the application of cavitation for wastewater treatment described in detail how ultrasound waves (acoustic cavitation), as well as pressure waves (hydrodynamic cavitation) in combination with other advanced oxidation processes (AOP’s) for wastewater treatment, have been proved to be advantageous for oxidising the organic pollutants as a result of synergistic effect.

Additionally, they demonstrated the effectiveness of various processes in oxidising the organic contaminants, dependencies on the process parameters, and suggesting the propitiousness of combining ultrasound technology with the AOP’s [16]. Further, Bethi et al., in their review reported the usage of ultrasound-assisted technology for developing nanomaterials which, when utilised with the AOP’s resulted in better treatment of the wastewater. This advantage can again be attributed to the synergistic effect caused by the hybrid intensified processes [32]. To further corroborate the synergism effect associated with ultrasound-assisted processes, Bhargava et al. highlighted the enhancements caused by ultrasound technology coupled with the conventional techniques [31]. In the pharmaceutical sector, sonocrystallization has attracted profound attention. Hussain et al. elucidated how ultrasound benefited the crystallisation of ASA-Asprin in terms of reduced induction time due to accelerated nucleation, although conditions involved low saturation levels [33]. Further Isari et al., in their studies on sono-photolytic degradation of pharmaceutical wastewater, exemplified the synergistic effect caused when ultrasound is coupled with the conventional techniques [34]. All these diverse research works based upon sonochemistry and synergism demonstrate this intensified technique's propitious applicability.

Hydrogen is one of the prime importance in the researcher’s quest for alternative energy sources. The main advantages of the extensive use of hydrogen are its abundance in the atmosphere, and it’s carbon-free emission property upon combustion [35]. Hydrogen is also the most abundant and lightest reactive gas, making it very economical to produce and manufacture if specific routes are employed [36]. Another unique feature of hydrogen is that it is an energy carrier unlike gasoline, coal etc. The energy content of hydrogen, specifically liquid hydrogen is higher than many other significant fuels like crude oil, coal and LPG. While hydrogen has an energy content of 120.94 MJ/kg [37], the other fuel’s energy content ranged from 20 to 50 MJ/kg [38]. This shows the significance of hydrogen for its use as a renewable energy source. Three aspects that need to be addressed for establishing hydrogen as a better alternative fuel are production, storage and usage.

Hydrogen is known for its enormous calorific value, and its eco-friendliness making it most preferred green energy alternative over the currently used fossil fuels if it is considered for long term application [39]. The only by-product of it is water which is quite valuable, and thus its applications are increasing in the fields such as transportation, portable electronics, etc. However, with many merits, few demerits also associated with hydrogen as an energy source such as the cost and problems associated with its production, storage and transport difficulties, etc. [40]. Thus, researchers focus on solving these problems by devising disparate technologies, methodologies, and pathways to produce, store, and transport hydrogen.

Many methodologies and sources are used for hydrogen production [41] such as electrolysis [42], thermolysis [43], photocatalysis [44], biophotolysis [45] and coal gasification [46]. Thus for its performance-evaluation process, simulation tools were used to identify the most promising pathway [47]. Steam reforming is proved to be the best fit owing to the ease of production, low production and operational cost and hence is extensively used for H_2_ production earlier. But at the expense of these advantages, the method shows a significant drawback of being non-ecofriendly. So, the scientific community attempts to develop clean and green production methodologies and come up with electrolysis, photocatalysis, biological photosynthesis, etc. Again, these methods didn’t show any favorable results in the form of efficiency. However, few studies involving photocatalysts [48] and metal-doped catalysts [49] have shown promising results in hydrogen production. Like other applications where ultrasound is proved beneficial, the cavitation phenomenon in water due to power ultrasound is promising for clean hydrogen production. Researchers have attempted to use ultrasound directly or indirectly for the techno-economically efficient hydrogen production and reported that sonolysis is better than other conventional techniques. Apart from that, it has also been found that ultrasound, when combined with other conventional clean methods like electrolysis, resulted in an overall increased hydrogen yield. All these studies have been compiled and described in this review. The main reason behind the advantages exhibited by sonolysis is due to the formation of H^•^, OH^•^, HO_₂_^•^ and O radicals which enhance the rates of reaction by many folds. Additionally, the turbulence created due to the propagation of alternating pressure waves enhances the mass transfer rates. The thickness of liquid films is reduced; thus, the gas transfer is enhanced, and bubble coalescence is reduced; thus, the interfacial area for gas transfer is increased [50].

On a large scale, hydrogen transport and storage have many associated challenges, and thus the development of robust, benign and cheap container material has become primarily important [51]. The material's key properties while determining its applicability for storage and transport containers are weight, cost, recyclability, and kinetics of adsorption and desorption [52]. Storage of hydrogen was earlier noted to be a prime challenge, especially since it is highly flammable and the rise in public awareness due to hydrogen-related incidents such as Hindenburg accident [53]. But introducing ultrasound is proved to solve the problems to a remarkable extent, which is expounded in this review. Hydrogen storage can be broadly classified as physical and material based [54]. The earlier physical methods include compressed gas [55], cryo-compressed hydrogen storage [56] and liquid hydrogen storage [57]. Whereas, the extensively reported material-based storage methods include chemical sorption using metal hydrides [58] and physical sorption mainly comprising of metal–organic frameworks [59] and electrochemical storage systems where the hydrogen generated in a redox reaction is directly physisorbed at the electrode [60].

**Fig. 2:**
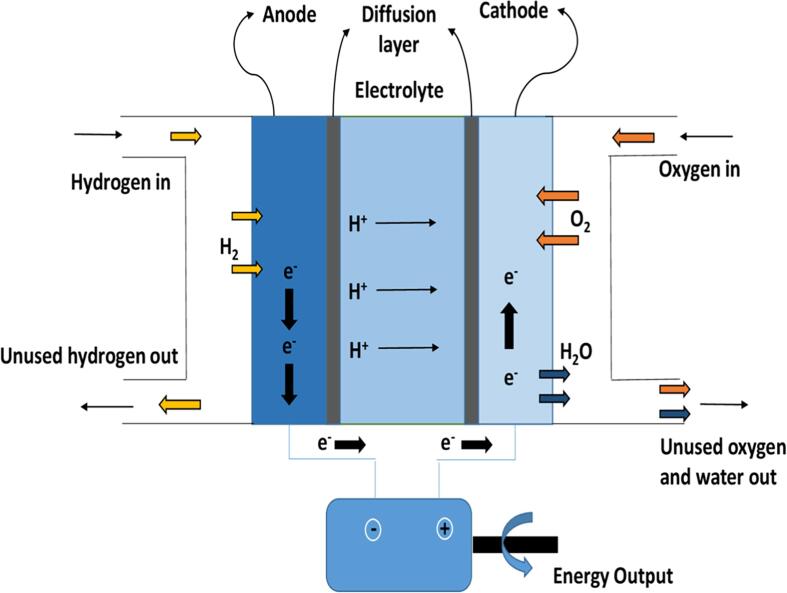
Block diagram of a hydrogen-based fuel cell.

In usage, the central area of hydrogen for energy production and usage is fuel cells. They have been utilised as power sources for both backup and primary type power generation at remote places, spacecraft, etc. and also as micro combined heat and power (micro CHP) systems for residencies and offices. Some of the revolutionary applications have been observed in electronics, electric automotive and portable power systems for small to large power generation. Many types of fuel cells [61] are existing such as solid polymer fuel cells (SPFC) [62], proton-exchange membrane (PEM) fuel cells [63], alkaline fuel cells (AFC) [63], phosphoric acid fuel cells (PAFC) [64], molten carbonate fuel cells (MCFC) [65], and solid oxide fuel cells (SOFC) [66]. Each fuel cell is advantageous and useful under certain temperature and pressure conditions. Hydrogen can be used as a fuel in all the fuel cells as indicated earlier but is most suitable for PEM fuel cells since it results in the production of very less amounts of carbon monoxide, hence lower poisoning of the catalyst [67]. The detailed summary has been given by Litster and McLean [68] and Wee et al. [69] on the fabrication routes of PEM fuel cells and catalyst used in their production. Fig. 2 demonstrates the working of a hydrogen fuel cell. It can be observed that on the anode side, hydrogen molecules divide into protons and electrons. The protons then pass through the electrolyte membrane while the electrons are forced through the circuit, producing excess heat and electricity. The electrons, protons and oxygen then combine on the cathode to form water molecule, making this whole process completely clean, and productive.

Apart from this, the application of hydrogen is extended to a range of industrial sectors, for example, ammonia production via Haber-Bosch process being at the top of the list. Glass industries, electronics, metal refining, etc. also utilise hydrogen. In habitat sustentation, the primary utilisation of hydrogen was observed in the refining of crude oil to generate untainted fuels by removing the contaminants, including nitrogen, metals etc., that are congenitally present in crude oil. Although refining could make the fuels more effective, the surge in the requirements of the ever-increasing population for energy has caused the scientific community to find an alternate source of fuel. Biofuels proved to be the best solution to solve this problem and follow the government initiatives of promoting environmentally friendly and sustainable energy sources. One of the major advantages that biofuels exhibit apart from being renewable, biodegradable and non-toxic is that the amount of carbon dioxide it generates is similar to that which is absorbed by the biomass source, i.e. plants via photosynthesis. Thus making it a carbon–neutral source of energy. The only problem faced by biofuels is that the conventional transesterification process used for the production is slow [70]. Over the years, the ultrasound-assisted route is gaining increasing interests in various research fields, more importantly, as a green and robust process intensified technique. Some of the applications of hydrogen too attracted its attention. Hydrogen-based fuel cells, hydrodesulfurisation, denitrification, and biofuels production are applications where ultrasound proved propitious.

In this review, the promising applications of sonochemical methods in hydrogen usage, production and storage have been explored and elucidated.

## 2 Sonochemical equipments

Ultrasound for acoustic cavitation is applied via probe and bath sonicators. Also, high intensity focused ultrasound (HIFU) is considered the most advanced, along with other techniques.

**Fig. 3:**
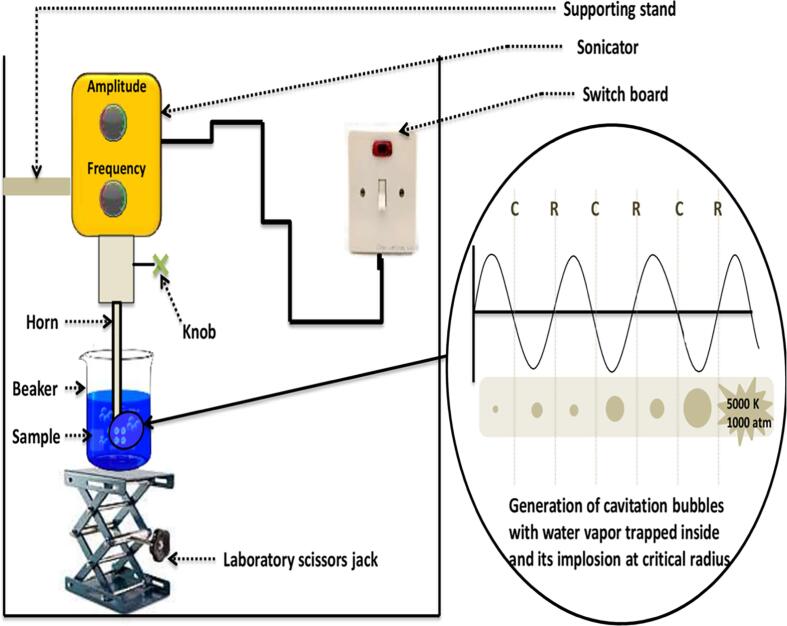
Schematic diagram of the sonoreactor and description of the cavitation phenomenon.

Acoustic cavitation: In short, it refers to nucleation, growth and collapse of the bubbles under acoustic waves. It is carried out either using a probe-type ultrasonic homogeniser (Fig. 3) or an ultrasonic bath. Both works on the principle of generating cavitation wherein high power ultrasound is introduced into a liquid medium causing the sound waves to transmit in the fluid and create alternating high-pressure (compression) and low-pressure (rarefaction) cycles, with the frequency-dependent rate of oscillation.

Small bubbles of the vacuum are generated in the liquid milieu due to high-intensity ultrasonic waves during the low-pressure cycle. With the generation of bubbles, they undergo alternating compression and rarefaction cycles, during which the volume of bubbles gradually increases. At the critical point, when the bubbles burgeon to a specific size, they unable to absorb more energy as a result of which, during a high-pressure cycle, they implode. The outcome of this implosion is the generation of very high temperatures (approx. 5,000 K), pressures (approx. 1,000 atm) in the nearby milieu as well as the generation of liquid jets the velocity of which has been found to reach as high as 1500 m/s [1]. Due to the abnormal local temperature and pressure maxima created by the transient collapse during ultrasonication of liquids, various effects have been identified and studied that include extraction, homogenisation, lysis (leads to valuable polyphenols from green tea leaves by breaking the cell wall) etc. which show myriad applications in disparate fields. Both the techniques mentioned above use ultrasound, yet the efficiency and effectiveness are different.

HIFU: It’s a non-invasive and continuous technique that works at a lower frequency and uses focusing effect of acoustic lenses/curved transducers on achieving the necessary intensity (high sound pressure) at the target placed at the focal point of the device. This therapeutic technique has numerous medical applications [71]**.**

## 3 Sonochemical methods vs conventional methods

In a sonochemical reactor, acoustic cavitation leads to various effects such as high shear stress near the bubble wall, high temperature and pressure in the milieu, production of microjets, etc. which provide the opportunity to use it for different applications and study the intensified processes [72]. Enhanced properties were observed in kinetics, selectivity, extraction, dissolution, and filtration due to the cavitation effect. Advantages are also seen in terms of enhanced electrical and mechanical properties. Sonochemical reactors can be more energy-efficient than the stirred vessel if designed and operated appropriately [73]. Caupin et al. [74] also reported that the chemical and physical effects caused by ultrasound are mainly due to the cavitation effects.

Using some of the following examples, the advantages of the sonochemical process compared to the conventional methods could be realised.

1. Ultrasound acts as a catalyst itself and doesn’t need to be replenished like a typical catalyst.
2. It is environmentally-friendly.
3. Sonochemical process involves using solvents that are cheap and renewable.
4. No requirement of extreme operational conditions.
5. Ceria nanoparticles were produced using both the sonochemical and conventional methods, and it was proved that the sonochemical route is better for the production due to the following [75]:
• Time-saving as its optimum reaction time was 20 min, whereas, in the conventional method, it was 4 h.
• Energy-efficient as it saved 92% of the energy.
• Increased yields and average particle size, which were due to rapid micromixing and thus faster reaction.
6. Enhanced extraction yields without decomposition of the material [76].
7. Higher morphological homogeneity [77].
8. High amorphicity of the products. Wang et al. [78] developed amorphous Fe3O4 nanoparticles (NPs) using an ultrasound approach and found a high surface area and excellent specific capacitance at room temperature. Apart from this, they noted amorphicity, which was favourable for many applications.
9. New reaction routes and no side reactions.
10. Enhanced polymer properties such as low curing temperature, high glass transition temperature, better mechanical properties, etc. [79].
11. Versatility: Just with slight modification in the reaction conditions, various nanostructured materials can be manufactured including metals, metal alloys, metal oxides, metal sulphides, carbides, nanostructure supported catalysts, carbon, polymers and biomaterials [80]. The reason behind this favourable property can be attributed to the enormous range of frequency, amplitude and intensity of ultrasound that can be employed. The optimised operational parameters employed for a particular application result in creating reaction conditions due to the cavitational effect. Further, high frequency can be utilised for degradation or breaking while the low frequency can be used for synthesis.

Although not every effect and result of ultrasound has been studied thoroughly, researchers have valid reasons for many observed effects. Some of them are:

1. Time/energy saving: This is because of the macromolecular mixing caused by acoustic irradiation and the generation of hot spots. As less time is required, the energy is also saved.
2. High yield and selectivity: Both physical and chemical transformations are altered because of the cavitation phenomenon, causing efficient agitation, dissolution, mass and heat transfers, and reagent sonolysis. The chemo-, regio- and stereoselectivity is enhanced, and thus, the overall selectivity is increased.
3. High reaction rates: The formation of H^•^, OH^•^, HO_₂_^•^ and O radicals causes the reaction rates to increase, leading to faster redox reactions. Additionally, the turbulence created due to the propagation of alternating pressure waves enhances the mass transfer rates. The thickness of liquid films is reduced; thus, the gas transfer is enhanced, and bubble coalescence is reduced; thereby, the interfacial area for gas transfer is increased [50].
4. High extraction yield: It is achieved because of site-specific cleavage or scission. Considering green tea as an example, ultrasound can be favourable to extract valuable catechins effectively [70].
5. Better morphological properties: The impact created by microjet and shockwaves has been known to substantially influence the chemical composition and physical morphology of the solids.
6. Amorphicity: The high cooling rate (10^11^ K/s), doesn’t let the product crystallise, which is beneficial as sometimes amorphous products are active than their corresponding crystalline counterparts [81]. For example, amorphous CoP electrocatalyst outperforms its crystalline counterpart for water splitting [82].

Many researchers reported on the sonochemistry-assisted synthesis of nanomaterials, and their results have proved that this route of synthesis is better than the conventional route in many cases (Table 1).

**Table 1:** Earlier studies on the synthesis of different materials using sonochemical methods as compared to the conventional methods.

References	Produced material	Synthesis route	Obtained results
[83]	Kaolin-chitosan-TiO_2_	Ultrasound-assisted in contrast to the hydrothermal method	Smaller particle size, higher surface area and enhanced adsorption capacity
[84]	Calcium carbonate	Reverse miniemulsion technique using ultrasound	Controlled particle size, uniform morphology and high conversion
[85]	Chitosan-ZnO-TiO_2_	Ultrasound-assisted method in contrast to mechanical stirring	Smaller particle size and enhanced adsorption capacity
[86]	Chalcone (3-(4-fluorophenyl)-1-(4-methoxyphenyl)prop-2-en-1-one)	Sonochemical route in contrast to the conventional method	Better crystallinity and energy and time-efficient
[87]	Zinc molybdate and zinc phosphomolybdate	Sonochemical route in contrast to the conventional method	Improved solute transfer rate, rapid nucleation and environmental-friendly
[75]	CeO_2_	Sonochemical route in contrast to the conventional method	Energy and time-efficient
[23]	Bismuth tungstate nanostructures	Ultrasound-assisted hydrothermal route in contrast to the hydrothermal method	Higher crystallinity, decreased particle size and better size distribution
[88]	Bismuth vanadate	Pulse ultrasound-assisted hydrothermal route in contrast to the hydrothermal method	Suitable properties for water splitting applications
[89]	HZSM-5 catalyst	Sonochemical route in contrast to the conventional method	Smaller particle size and higher crystallinity in less time
[90]	Barium titanate	Sonochemical method in contrast to the mechanochemical approach	Powerful and feasible at a much lower processing temperature
[91]	Valorisation of baobab seeds	Sonochemical route in contrast to the conventional method	Significant high recovery of TFC, TPC and antioxidant activity

Nevertheless, there are few disadvantages in the sonochemical process over other methods, mainly due to sudden bubble collapse. Hopefully, future research could help address these disadvantages from sudden and uncontrolled bubble collapse, which pose undesirable effects.

## 4 Hydrogen production

Hydrogen due to its advantages, namely, high specific energy, abundance, green, etc. has been proved to be the fuel for the future. Thus, the scientific community is attempting to develop robust, techno-economically viable pathways for its production. The conventional methods used for the production of hydrogen are:

• Thermochemical [92]
• Electrochemical [93]
• Photobiological [94]
• Photoelectrochemical [95]

Out of these conventional techniques, hydrogen production using steam reforming, gasification, and autothermal reforming has been based on fossil fuel consumption. Surprisingly, >96% of H_2_ demand is met using these environmentally unfriendly methods [96]. Either these conventional techniques face the problem of being non-environmental friendly, or at the expense of becoming environmental-friendly, their scope for scale-up has been known to be hampered. Thus, there is a need to develop/incorporate new, advanced technologies for the benign production of hydrogen on an economic scale. Suppose the production and storage of hydrogen are made more efficient, due to the advantages of being green and possessing the highest calorific value, they can solve the problems of fuel required for the increasing population. In Table 2**,** the experimental parameters of various methods used to produce hydrogen and their respective efficiencies have been indicated. The production of hydrogen following sustainable, benign and techno-economical pathway is the only solution for global warming, transportation and energy security-related problems.

**Table 2:** Summary of hydrogen produced using different techniques and their performances.

References	Method used for the production	Chemical reactions	Experimental parameters and the obtained results
[97]	Steam reforming	CH_4_ + H_2_O CO + 3H_2_ ΔH = 206 kJ/mol CO + H_2_O CO_2_ + H_2_ ΔH = -41 kJ/mol Temperature > 500 ^0^C	Natural gas in REP - Cost= $3.71/kg H_2_
[98]	Autothermal reforming	2CH_4_ + O_2_ + CO_2_ → 3H_2_ + 3CO + H_2_O 4CH_4_ + O_2_ + 2H_2_O → 10H_2_ + 4CO	For large scale production η = 7–74% , Cost=$1.93 /kg H_2_
[99]	Biomass gasification	C_6_H_12_O_6_ + O_2_ + H_2_O → CO + CO_2_ + H_2_ CO + H_2_O → CO_2_ + H_2_ Temp (>700 ^0^C)	η = 72% Cost=$5.14/kg H_2_
[97]	Electrolysis	Cathode: 4H^+^ + 4e^-^ = 2H_2_ Anode: 2H_2_O = O_2_ + H_2_ + 4e^-^ (Burning of fossil fuels)	η ≅ 25 PEM- $5.14/kg H_2_ SOEC- $4.96/kg H_2_
[100]	Thermolysis	H_2_O → H^o^ + OH^o^ OH^o^ → H^o^ + O H^o^ + H^o^ → H_2_	For the capacity of 172 GJ η = 4.11% Cost=$68 G/J
[101]	Photoelectro- -chemical	One-step method, which works on the phenomenon of water-splitting that uses sunlight irradiation on the water-immersed semiconductor	For 50,000 kg H_2_/day Photoelectrode system: η = 15% Cost=$17.30 /kg H_2_ DBPS system η = 1% Cost=$28.60 /kg H_2_
[102]	Biophotolysis	CO_2_ + H_2_O → 6(CH_2_O) + O_2_ Light energy → Biochemical energy (during the photochemical reaction) Uses microalgae or cyanobacteria and hydrogenase or nitrogenase enzymes, the biochemical energy is converted to hydrogen.	For 50,000 kg H_2_/day η = 4.5% Cost=$9.20/kg H_2_ (Estimated target for the year 2020 by DOE)
[98]	Pyrolysis	Biomass → H_2_ + CO + CO_2_ + GH Heated in the absence of air, GH’s are the gaseous hydrocarbons produced.	For midsize production scale η = 56% Cost=$1.21–2.19/kg H_2_
[103]	Dark fermentation	Organic matter → Biohydrogen In the dark, the presence of anaerobic bacteria and enzymes; photo fermentation occurs in the presence of light.	For the production output of 50 kg H_2_/day Cost > 50$/Kg
[104]	Ultrasound-based	Mechanism is indicated in Equation (1)	Max. efficiency − 96% Production efficiency − 78%

Ultrasound promises to be a prosperous and secured solution for the environmentally benign production of hydrogen, without requiring any catalysts or employing high temperatures or high pressures. The earlier studies have proved to be a better technique for hydrogen production compared to other conventional methods. It is also evident from Table 2 that ultrasound-assisted production of hydrogen outperforms other conventional methods. It has been reported that the yield of hydrogen produced using ultrasound is 200 times compared to photocatalysis [105]. When imparted into liquid water, the high-frequency ultrasonic waves (20–1000 kHz) can dissociate the water molecules and produce hydrogen more efficiently due to cavitational effects [106], [107]. The chemical reaction occurring during sonolysis is described in **Equations** (1.1), (1.2), (1.3), (1.4), (1.5) [108], [109], [110], [111]. Due to the heating effect caused by cavitation, the dissociation of the water molecule takes place, whereas, during the cooling period, the association of hydrogen radicals takes place [112].

(1.1) H_2_O + ultrasound → OH^•^ + H^•^

(1.2) OH^•^ + H^•^ → H_2_ + O

(1.3) OH^•^ + OH^•^ → H_2_O_2_

(1.4) H_2_O_2_ → H_2_O +½ O_2_

(1.5) H^•^ + OH^•^ → H_2_O

As the function of ultrasound equipment depends on various factors, hydrogen production has also shown dependency on those factors. Koda et al. [113] investigated the acoustic field elements, the equipment's efficiency, rates of sonochemical reaction, frequency, the intensity of ultrasound and dissolved gas. The specific factors affecting the hydrogen production rate are yet to be understood. Still, for the enhancement of production of hydrogen, it can at least be suggested to use optimised ultrasound frequency (20–1000 kHz) [101], [102], [103], [104], high acoustic intensity and a dissolved gas with high heat capacity and low thermal conductivity [27], [99], [107], [108], [109]. With the advantages of ultrasound in hydrogen production, researchers started developing pathways to use it while considering the constraints such as yield, environmental friendliness, and cost. Although not much research was initially seen to be focused on the ultrasound-based synthesis of hydrogen, since 2015, there has been a surge in the research activities in this field. Merouani and Hamdaoui have reported in detail the research works accomplished till 2015 [108].

One of the recently developed ultrasound-based hydrogen production methods is “The Sono-Hydro-Gen process” which is a green alternative compared to conventional techniques [106]. A variety of hydrogen production techniques exist with each having their pros and cons associated with production. Dincer et al. [110] Islam et al. [111] and many others have described the methods, challenges and opportunities associated with those methods in detail. Bruno Pollet is the pioneer researcher who tried to meticulously explain sonication involvement in heterogeneous electron transfer kinetics, which helps achieve increased hydrogen evolution reaction (HER) by reducing the overpotential and ohmic drop [112], [113], [114]. In this review, the recently (2013–2020) accomplished research studies in hydrogen production utilising ultrasound technology are analysed directly or indirectly. The considered operating parameters during the production have also been shown in Table 3**.** The major focus is given to parameters, namely, frequency, intensity, static pressure, liquid temperature, pH and bubble size.

**Table 3:** Recently reported studies on the production of hydrogen with direct or indirect utilisation of ultrasound.

Reference	Accomplished study	Operational parameters	Key results
Direct use of ultrasound			
2013 [127]	Ultrasound-assisted production of hydrogen using NH_3_BH_3_ and Co-B catalyst.	Frequency = 35 kHz Amount of catalyst = 0.005 gm Temp = 80 ^0^C	Maximum hydrogen generation = 9157.20 ml.min^−1^.gcat^−1^ (37.79% more than magnetic stirring). Activation energy = 47.50 kJ.mol^-1^ Catalyst reusable even after four turns.
2014 [104]	Ultrasound coupled with alkaline water electrolysis	Frequency = 20 kHz Amplitude = 30% Temp = 25 ^0^C	Hydrogen production efficiency = 78% (increased by 4.5%). Electrode surface area was increased by 43.75%0. 10–25% energy savings and energy efficiency increased by 1.3%.
2015 [122]	Ultrasound-assisted production of hydrogen using water	Frequency = 20–1100 kHz Intensity = 0.5–1 W/cm^2^ Temp = 20–50 ^0^C Gases for saturation - Argon and air	Hydrogen production was more at: • Lower frequencies • Higher intensities • 30 ^0^C • Argon gas Bubble radius decreased with frequency
2015 [128]	Hydrogen production from water using photocatalysis coupled with ultrasound	Catalyst - Y_0.8_Ga_0.2_InO_3_ doped with sulphur atoms (0.4 g). Frequency = 38 kHz Argon gas, 25 ^0^C, 1 bar	Only through photocatalysis H_2_ produced = 1 μmol in 1 h. Only using ultrasound H_2_ produced = 80 μmol in 1 h. Using photocatalysis and ultrasound together, H_2_ produced = 125 μmol in 1 h.
2017 [129]	Hydrogen production from the water via ultrasound coupled photocatalysis	Catalyst - reduced graphene oxide-based CdS (1 g) Frequency = 20 kHz	Ultrasound coupled photocatalysis led to hydrogen yield of 8 ml, whereas just photocatalysis resulted into 4.8 ml. Max. production was at 35 W. With an increase in temperature from 25 to 45 ^0^C, the production increased by 70%.
2018 & 2019 [130], [131]	Hydrogen production from water using ultrasound coupled with laser-ablated metals	Metals used - Al, Mg, Ti, Si, and Al-Mg alloy Frequency = 40 kHz Power = 70 W	Hydrogen yield of 1300 ml/min per gram of aluminium was reported in 2018. Hydrogen yield of 23.2 mmol/min per gram of Mg_2_Al(OH)_7_ nanosheets was reported in 2019.
2020 [132]	Ultrasound (US) coupled dark fermentation, and heat shock (HS) route was used for the synthesis of hydrogen	Frequency = 40 kHz Temperature = 20 ^0^C Ultrasound specific energy (USE) = 20.5–102.5 kJ.L^−1^ Heat shock parameters = 45 min, 85 ^0^C	Optimised USE for anaerobic sludge treatment = 41–61.5 kJ/L when only US was used, 61.5–82 kJ/L when US was used along with HS. Only US and US + HS had 79.5% and 19.6% more H_2_ yield, respectively than HS.
2020 [133]	Acceleration of hydrogen production using Al-Ga-Gr material and ultrasound	US power = 180–216 W	Conversion rate = 100% in 20 min. 80% yield reached in 300 s using US. Max. production rate = 1582 ml/min/g Al, and mean hydrogen rate was 2.5 (at 180 W), and 3 (at 216 W) folds more than that reported when US was not used.

Indirect use of ultrasound			
2013–2017 [134], [135], [136]	Ultrasound utilised to obtain catalyst for its application in reforming for hydrogen production	CO_2_ reforming of methane using Ni/ZSM-5 nanocatalyst; Parameters of US − 20 kHz, 90 W, 45 min	Ni (8% (wt/wt))/ZSM-5 at 850 ^0^C reported highest yield − 67% H_2_ and 80% CO, and the conversion was 82% CH_4_ and 80% CO_2_. Stability for 24 h was reported. The particle size of Ni of 99% was less than 100 nm.
		CO_2_ reforming of methane using Ni/ZSM-5/ZrO_2_ nanocatalyst Parameters of US − 20 kHz, 90 W, 45 min	Ni (8%)/ZrO_2_ (5%)-ZSM-5 at 750 ^0^C reported higher conversion of CH_4_ to CO_2_ than that of Ni (8% (wt/wt))/ZSM-5 at 850 ^0^C. At 850 ^0^C, yield of CO = 95% and H_2_ = 90%. Stability for 600 min was reported.
		Steam reforming of ethane using CoMgAl as the catalyst Parameters of US − 42 kHz, 65 ^0^C, 1.5 h	The catalyst with 10 and 15% Co reported the highest H_2_ production. 3.82 mol hydrogen/mol ethanol was reported.
2017–2019 [137], [138], [139], [140], [141], [142]	Ultrasound utilised to obtain photocatalyst for its application in photocatalytic hydrogen production via water splitting	Photocatalyst - Cu_2_O Microwave (150 W) and Ultrasound (150 W) Temperature – 80 ^0^C	In the presence of glucose as the reducing agent max. hydrogen yield was 400 µmol/g of catalyst in 3 h. Cu_2_O preparation time − 1 h
		Photocatalyst- TiO_2_ dispersed on Clinoptilolite Parameters of US − 24 kHz, 200 W	Max. hydrogen yield was 569.88 µmol/g catalyst in 1 h, was 8 folds more than that obtained using reference TiO_2_ sample. The obtained photocomposite showed reusability.
		Photocatalyst-reduced graphene oxide (rGO) incorporated InVO_4_-TiO_2_ Parameters of US − 20 kHz, 750 W, 80 ^0^C	Max. hydrogen yield was 1669 µmol/h.
		Photocatalyst- MoS_2_/CdS Parameters of US − 40 kHz, 150 W, 30 ^0^C	Max. hydrogen yield was 15260 µmol/g/h using 1.5 wt% MoS_2_/CdS.
		Photocatalyst- Cd_x_Zn_1-x_S (x = [0,1]) Temp = 70 ^0^C	Max. hydrogen yield = 12 × 10^-4^ mL/mg/min and quantum yield of 1.4%.
		Photocatalyst - Ca_3_MnO_6_ was synthesised using conventional processes, ultrasound and microwave	Ca_3_MnO_6_ synthesised using ultrasound and microwave coupled possessed better morphology, purity, efficiency, high turnover, and frequency than synthesised using only microwave.
2017 [143]	Biohydrogen production using fermentation method and ultrasound pretreated waste activated sludge	Acetate-type fermentation Frequency = 40 kHz Temp = 36 ^0^C Power = 20 W/L	Biohydrogen yield of 68.9 per gram of soluble chemical oxygen demand (SCOD) of the pretreated sludge. SCOD/(total COD) obtained after ultrasound treatment was 20.2%.
2017 [144]	Biohydrogen production from effluent wastewater using fermentation method and ultrasound pretreated Rhodobacter sphaeroides	Photofermentation Frequency = 20 kHz Amplitude = 15/ 30/ 45 % Time = 5/10/15 min. Temp = 30 ^0^C	The highest yield of 9.982 ml H_2_/ mL was reported in 10 min when amplitude was 30%. COD removal was also found to be higher during this process.
2018 [145]	Ultrasound coupled dilute acid pretreatment of grass for biohydrogen production via fermentation	Batch hydrogen fermentation Dilute HCl US power = 260 W	Treatment increased the SCOD and soluble carbohydrate contents of grass by 98.6 and 236.9%, respectively. Biohydrogen yield of 42.2 ml/gm of grass in 30 min was 311.7%, which is more than when the pretreatment was not carried out.
2020 [146]	Ultrasound coupled alkali pretreatment of hazardous antibiotic fermentation residues for fermentation-based biohydrogen production	Batch hydrogen fermentation Alkali - NaOH Frequency = 20–25 kHz US power = 400 W	Treatment increased the SCOD and soluble carbohydrate contents of grass by 61.6 and 105% respectively. Biohydrogen yield of 17 ml/g of volatile solids in 30 min was 78.9% more than when the pretreatment was not done.

Since many researchers employ similar operational parameters, it can be concluded that hydrogen evolution is more at lower frequencies (20 kHz) which reduces with an increase in frequency. With an increase in frequency, although the number of bubbles increases, they implode quickly resulting in the formation of radicals instead of hydrogen gas [107], [108]. Considering ultrasound intensity, the higher intensity has proved to be propitious. It causes higher bubble collapse temperature, collapse time, more amount of water trapped in the bubbles, and the number of radicals [115], [116], [117], [118], [119], [120], [121]. It has been found that in the presence of an inert gas such as argon, the yield was more as compared to when the air was used [122]. For liquid temperature and pressure, 20–30 ^0^C and atmospheric pressure were mostly used. Temperatures above 30 ^0^C were reported not beneficial as it leads to bubble collapse to occur at a lower temperature, causing the formation of a lower number of radicals. In contrast, pressures above 1 atm led to lowering the effect of intensity.

Additionally, Merouani and Hamdaoui [123] reported on the optimum acoustic bubble temperature (3500±200 K) and pressure (100±10 atm). Bubble radius was reported to be dependent on frequency, intensity and liquid temperature [118]. Merouani and Hamdaoui reported the optimum bubble size as 4.8 µm at 355 kHz and 1 W/cm^2^ [124]. Lastly, for the indirect utilisation of ultrasound to produce materials like the catalyst for photocatalysis or reforming, the concentration of catalyst used also played a significant role.

Many researchers have coupled ultrasound with conventional methods and found an enhancement in production. This can be attributed to the reduction in electrical, electrochemical, transport-related and other resistances used to get involved during the production and gradually reduce the efficiency of the process [104]. Some of the highlighted processes incorporating ultrasound indirectly for hydrogen production proved to be favourable, including reforming, photocatalysis, and fermentation. In Table 3**,** the recent research works in this context are also indicated.

Reforming, a promising route to obtain synthesis gas using methane and carbon dioxide as starting materials, has attracted researchers' attention. Methane and carbon dioxide being greenhouse gases, making the process environmentally acceptable, although one of its product carbon monoxide makes it non-ecofriendly. Additionally, the requirement of higher temperature causes a problem. The heterogeneous nanostructured catalysts have found their application in dry reforming and steam reforming to solve instability, the requirement of high temperature and low yields. Although the catalysts could solve such the earlier issues, they have other issues such as agglomeration, low catalytic activity, etc. Ultrasound-assisted synthesis of these catalysts overcome such issues. The catalyst synthesised via ultrasound route showed reduced particle size, better particle distribution, surface area, morphology and performance, which led to many valuable materials.

Photocatalytic water splitting has been a sustainable hydrogen production route from solar energy, which requires a semiconductor material with its conduction band edge slightly more negative than the potential required to reduce H^+^ to H_2_. Various metal oxides have been utilised in the preparation of photocatalysts, mainly, Ti or Cu based. However, the problem faced with pure oxides is that they exhibit poor activity and conversion rate. This is due to quick re-amalgamation of photo-generated charge carriers, a huge overpotential requirement for the production of hydrogen, and a high potential barrier to the water-splitting process. This could be solved through synthesising mixed metal oxides, binary/ternary composite, and even using the ultrasound-assisted route. Ultrasound promotes photocatalysts' formation with favourable properties such as monodispersity, smaller particle size, proper positioning of conduction and valence bands, reusability, high surface area, etc. Ultrasound leads to more active sites and larger surface area; thereby, more water can be absorbed, hence intense reduction, causing an increase in the hydrogen yield. Additionally, studies also advanced from photocatalysis to electrocatalysis and even photoelectrocatalysis [125], [126].

Biohydrogen, an alternative similar to biofuel, has been suggested as an alternative to fossil fuel. It was initially produced using environmentally friendly photofermentation process, but it was required to incorporate a better technology for the enhanced yield and economic viability. The reason behind obtaining low yield was the photofermentation pathway where the rigid lignin complex structure caused resistance. Various chemical, physical and combined methods have been suggested out of which ultrasound is proved to be the best. Low-frequency ultrasound, in the presence or absence of an acid, is used for enhancing the fermentation process by stimulating the bioactivity of abundant biomass such as waste sludge and residues and result into better hydrogen production from clean water or industrial wastewater. Thus, combining ultrasound with the previously used conventional techniques proved beneficial through synergistic effects, significantly increasing overall efficiency.

## 5 Hydrogen storage using sonochemically synthesized material

With an increasing demand for renewable energy, research on hydrogen utilisation is becoming more important. The one crucial bottleneck for the pragmatic approach in using hydrogen as a fuel is hydrogen storage. The approach to this problem is to devise compact, efficient systems and have high specific storage capacity. Material based H_2_ storage is picking up momentum in the past few years. Application of sonochemical methods for synthesis of these storage materials is a growing research interest. The application of the sonochemistry approach for hydrogen storage material carries various advantages. The resonant cavitation bubbles act as a media for reagent agitators, increasing the contact area between the reagents and increasing the surface area of the resulting products as well [147]. The main three types of material based storage systems are chemical, physical, and electrochemical, described in detail by Ramin et al. [53]. In the storage by the physical method, the hydrogen molecule is embedded in the metal structure by dissociating it into molecular form in the presence of a suitable metal [148]. Contrarily, in the physical storage systems, the required hydrogen for storage is physically embedded in the voids/spaces in suitable materials. The materials which exhibit these metal storage properties are metal–organic frameworks (MOF) and porous carbon materials [149]. Properties such as surface area, binding energy, charge and discharge kinetics are essential attributes that decide the throughputs of both these types of hydrogen storage systems [149]. Previous research has shown increased surface area, and enhanced surface properties for catalysts synthesised by the sonochemical methods. Thus this favorable outcome of the ultrasound-assisted mechanism is exploited to synthesise hydrogen storage material, as discussed further.

Previous studies have been carried out on utilising ultrasound to synthesise physical and chemical storage systems for the high capacity to store hydrogen. The facile fabrication and cost of synthesis make ultrasound suitable for the production of these storage materials. Farnoosh et al. [150] reported the synthesis of ZnO nanostructures using ultrasound and indicated its possible usage as a hydrogen storage system. Gang et al. [151], showed the sonochemical synthesis of copper oxide nanorods and reported high hydrogen storage ability of 165 mAh/g in terms of electrochemical hydrogen storage. They also reported a discharge capacity of 503 mAh/g that corresponds to 1.84% of hydrogen. Gang et al. [151] proposed the following electrochemical storage mechanism, as suggested by other researchers[152], [153]:

(1) CuO nanorods + H_2_O + e^−^ → CuO nanorods/H_surface_ + OH^−^

(2) CuO nanorods/H_surface_ → CuO nanorods/H_interstitial_

Peng et al. succeeded in the synthesis of copper oxide for hydrogen storage [154]. They followed a precursor dehydration route, a process very different than the sonochemical method, based on the same electrochemical hydrogen storage mechanism and reported a similar discharge capacity as indicated by Gang et al. [151]. This is a good example of how the sonochemical synthesis approach can yield similar or the same storage and discharge capacities as other chemical approaches.

Apart from nanotubes, researchers have investigated the usage of metal–organic frameworks (MOFs) for hydrogen storage. Surface area is a vital attribute to determine the hydrogen storage capacity, which demonstrates a direct linear relationship. Mohammad et al. [155] investigated the adsorption surface area of Cd(II) based MOF TMU-7 for gas storage, including hydrogen, with two synthesis methods, i.e. sonochemical and mechanochemical. TMU-7 synthesised by the mechanochemical method led to a BET surface area of 243 m^2^/g and synthesised by the sonochemical approach resulted in a surface area of 393 m^2^/g. This displays more increased storage capacity through the sonochemical based method compared to the mechanochemical method.

Electrochemical storage is another emerging and increasingly used method of hydrogen storage [150], [151], [152], [153]. This popularity for electrochemical storage has attributed the ease of hydrogen storage process since it does not require high pressures and adsorption process step as the hydrogen generated from an aqueous medium directly physisorbed at the electrode [160]. As described earlier, the electrochemical storage is facilitated by the redox reactions at the electrode materials; hence, the morphology and catalytic properties are of utmost importance for efficient electrochemical storage [160]. Previously materials for the electrochemical storage of hydrogen were synthesised using different methods, including thermal decomposition [150], [152], [155], [156], [157], [158], [159], [160], ultrasonic synthesis [167], combustion [159], [160] and chemical precipitation [162], [163], [164], [165], [166], [167], [168]. Maryam et al. [167] demonstrated an ultrasound facilitated synthesis of CdSnO_3_-graphene nanocomposites for the electrochemical storage of hydrogen. This work reported a hydrogen storage capacity of 2550 mAh/g after 20 cycles which increased from 690 mAh/g in the 1st cycle at 1 mA. This shows the efficacy of ultrasonic methods in the preparation of materials for the electrochemical storage of hydrogen.

Salehabadi et al. [159] described the electrochemical storage mechanism of hydrogen in Sr_3_Al_2_O_6_ nanostructure during charge and discharge cycles. The redox reaction occurring at the electrodes enables the storage of hydrogen. In their study, KOH was used as an electrolyte and Cu(OH)_2_ as the counter electrode. The working electrode material was nanostructural Sr_3_Al_2_O_6_. In the charging cycles during the galvanostatic discharge electrochemical measurements, the material at the working electrode was reduced **(Eq.** (2.1)**),** and at the counter electrode, it was oxidised **(Eq. B)**. As proposed by them, the complete charge cycle is given by **(Eq. C)** [159]. Table 4 illustrates a consolidated summary of significant research reported in hydrogen storage methodologies and their respective storage attributes.

(2.1) **Working electrode:** Sr_3_Al_2_O_6_ + nH_2_O + ne^-^ ↔ (Sr_3_Al_2_O_6_ + nH) + nOH^–^

(2.2) **Counter electrode:** Cu(OH)_2_ + OH^–^ ↔ CuOOH + H_2_O + e^-^

(2.3) **Overall redox reaction:** nCu(OH)_2_ + Sr_3_Al_2_O_6_ ↔ nCuOOH + (Sr_3_Al_2_O_6_ + nH)

**Table 4:** Summary of previously reported studies on the mechanisms of hydrogen storage using physical, chemical and electrochemical methods.

Reference	Synthesis methods	Compound	Storage attribute
*(i) Metal-Organic Frameworks (MOFs)*			
[169]	Solvothermal method	MOF-5	BET surface area of 839 m^2^/g was reported using Et_3_N as a solvent
[155]	Sonochemical synthesis	TMU-7	BET surface area of 393 m^2^/g was reported
[170]	Sonochemical synthesis	MOF-5	Langmuir surface area of 3208 m^2^/g was reported
[171]	Microwave synthesis	MIL-101	BET surface area of 3891 m^2^/g was observed
[172]	Electrochemical method	Cu_3_(BTC)_2_	Langmuir surface area of 1150 m^2^/g was reported
[173]	Mechanochemical methods	Cu_3_(BTC)_2_ [HKUST-1]	BET surface area of 278 m^2^/g was reported

*(ii) Polymer and Metal nanostructures*			
[174]	Doping-undoping-redoping route	Polyaniline and polypyrrole	6–8 wt% H_2_ sorption
[175]	Hypercrosslinking route	Polyaniline nanostructure	BET surface area of 20–632 m^2^/g and H_2_ storage capacity of 2.2 wt% were reported
[151]	Sonochemical route	Copper oxide	A hydrogen storage capacity of 1.84% was reported

*(iii) Chemical Hydrides*			
[176]	Solvent mediated milling	Ti-doped NaAlH_4_	A storage capacity of 4 wt% was observed
[177]	Mechanical milling	LiBH_4_ + 1/2 MgH_2_, 2–3 mol% of TiCl_3_	A reversible storage capacity of 8–10 wt% of H_2_ was reported
[178]	Sonochemical route	MgH_2_-Fluorographene	H_2_ uptake of 6 wt% was reported

*B. Electrochemical storage mechanism*			
[156]	Thermal decomposition	ZnAl_2_O_4_	Hydrogen storage capacity in terms of peak discharge capacity was 4000 mAh/g
[167]	Ultrasonic synthesis	CdSnO_3_-Graphene nanocomposite	Peak discharge capacity was 2550 mAh/g at 1 mA
[159]	Combustion route	Sr_3_Al_2_O_6_	Discharge capacity was 2500 mAh/g after 15 cycles
[168]	Chemical precipitation route	Zn_2_GeO_4_/graphene nanocomposite	The discharge capacity of the electrode reached 2695 mAh/g after 29 cycles
[161]	Thermal decomposition	Co_3_O_4_-CeO_2_ nanocomposite	Discharge capacity was 5200 mAh/g after 20 cycles

## 6 Hydrogen usage incorporating ultrasound technology

Hydrogen is present in abundance in the atmosphere, and in terms of energy, it is superior too, and hence its utilisation is very important. The Haber-Bosch process used for the mass production of ammonia is known to be one of the profound applications and the most important single use of hydrogen which brought a revolution in the agricultural field through the fertilisers. Both in industry and ecological conservation, hydrogen has been found to possess numerous applications. In ecological conservation, the primary utilisation of hydrogen is to remove sulphur, congenitally present in the fuels to generate untainted fuels. Using hydrogen for removal of sulphur assists in two ways: Firstly, making the fuel cleaner and secondly, it avoids the need for combustion, which causes the production of harmful sulphur oxide gases. Instead, desulphurisation using hydrogen produces H_2_S as the by-product, which can then be reacted with oxygen to give sulphur, which is the raw material in various industries [179], [180]. Apart from desulphurisation, hydrogen has been utilised to hydrogenate organic, nitro and aromatic compounds for numerous applications.

Hydrogen assimilated with a fuel cell is a promising benign energy source. Electricity is generated by the hydrogen-based fuel cell, which enables a vehicle to run cleanly and quietly. Inside an electric engine running on a fuel cell, hydrogen and oxygen are used as the chemical reactants that produce only energy and water with “zero emissions”, making it very environmentally sound. Researchers have not just stopped applying hydrogen-based fuel cells in vehicles but are trying to use it in aeronautics and maritime [181]. With similar motivation, Zodiac Aerospace, Dassault Aviation in collaboration with the CEA, Air Liquide are advancing in their project - Hycarus. They are working on using energy generated from fuel cells for the peripheral parts of the aircraft.

Similarly, in the marine sector, Energy Observer, working on Hycarus successfully developed the first-ever hydrogen vessel, wherein hydrogen was produced out of the ample seawater [182]. The proton-exchange membrane fuel cell (PEMFC) and solid oxide fuel cell (SOFC) are the most promising fuel cells for maritime applications [183]. Apart from that, in the space industry, hydrogen has been utilised as rocket fuel; and has also been used in fuel cell electric buses (FCEB), electric locomotives and fuel cell-based material transport vehicles [178], [179], [180].

Research has also been conducted to develop stationary fuel cells, which, by supplying electricity and heat, can be installed at houses, stores, etc. [184], [185], [186], [187]. Such systems are referred to as micro or mini combined heat and power (micro/mini CHP), wherein heat is collected as the by-product of the CHP and used to meet the demands [188]. Iain and Richard showed that domestic PEMFC and SOFC cost $ 25,000/kW. Still, it could be reduced to $1,000/kW if every house starts installing hydrogen fuel cells, thereby making it economically viable in the future [189].

In industries, hydrogen being a reactive gas has again found many applications. As a gas that could transfer active gases, its application in electronics offers excellent protection against oxidation and impurities [190]. In the glass industry, to manufacture flat glass using the float process, hydrogen has been used to provide the protective atmosphere [191]. As a strong reducing agent, it has been utilised to extract metals from their metal ores/oxides.

Out of the different usages of hydrogen in the industrial sectors, few attracted the attention of ultrasound-science. Researchers have started incorporating it to make that specific application more robust and techno-economically efficient. One such example is hydrogen-based fuel cells which are known to be the next-generation sustainable energy sources. In 1966 Bodine first reported in a patent, incorporating sonic energy in hydrogen-based fuel cell application [192]. In 2008–2009, Pollet reported utilising ultrasound to prepare PEMFC electrode material that exhibited enhanced electrochemical properties [193], [194]. Following this, the research in this field started gaining profound attention to the scientific community. Also, the refining of crude oil received intense attention owing to the importance of making the process environmentally sound. The commencement of research in this area can be dated back to 1951 when Barlow tried to convert hydrocarbons using catalysts and sonic vibrations. More studies have started focusing on this area [195]. The breakthrough occurred in 1993 when Lin et al. [196] elucidated about utilising ultrasound and surfactants together to convert asphaltenes to gas oil and resins efficiently. After this, in 2002, Yen et al. [197] described the effectiveness of ultrasound in the oxidative desulphurisation of fossil fuels, which brought a paradigm shift in the way researchers used to tackle the problem of sulphur contaminated fuels. Additionally, ultrasound-induced bioprocesses such as biofuel production, which is generated from biomass, assist in meeting the escalating transportation fuel needs and receiving intense attention. In 2000, Aliyu and Hepher reported the beneficial effects of ultrasound coupled with the enzymatic approach in the production of glucose, a precursor of biofuel [198]. After this, Colluci et al. [199] reported the feasibility of ultrasound mixing in biodiesel preparation from soyabean oil following the alkaline transesterification route. The conversion was 99.4% in just 15 min of ultrasound application which was much higher than that reported for mechanical agitation. With ultrasound technology being proved to be advantageous to the scientific community in the field of hydrogen-based fuel cells, refining of fuels and biofuels, researchers are working to make the application even better in the form of efficiency and benignness.

### 6.1 Fuel cell

Fuel cell is an energy conversion device, similar to an ordinary dry cell, with a cathode, anode and an electrolyte connected in an electric circuit. Owing to its advantages such as high energy density, high power density, cyclic stability, better thermal and mechanical stability, etc., in recent years the research on fuel cells has increased, and attempts are continuously made to make its production economically viable and environmentally friendly. Several developments have been observed to increase the productivity of a fuel cell such as incorporating electrocatalyst material in the electrode material of the fuel cell [195], [196], [197], polymer electrolyte membrane fuel cell [203], carbon nanotube-based fuel cell [199], [200], microbial fuel cell [201], [202], direct formate fuel cell [204], [205], [206], [207], [208], etc. The application of Pt-based electrocatalysts in fuel cells has attracted intense attention due to the high surface area it offers and environmentally-friendly nature. Various methods are utilised to produce these electrocatalysts, and the sonochemical approach has proved to be superior to them in terms of the fuel cell's electrochemical performance. Some of those research works and their results are shown in Table 5. Also, in Table 6, different primary methods for the preparation of Pt-based catalysts have been compared. The reason behind the sonochemical method that demonstrates better results is due to the synergistic effect. Pollet reported using ultrasound-assisted methods to prepare electrode material and electrocatalysts and elaborated the promising effects of cavitation and water sonolysis, which resulted in unique and favourable conditions in the milieu [209].

**Table 5:** Summary of Pt-based electrocatalysts synthesised using different methods and reaction conditions and their performance.

Ref. No.	Author(s) and Year	Electrocatalyst	Preparation method	Particle size (nm)	Electrochemical orFuel cell performance
[213]	Show and Ueno (2017)	Pt/CB	In-liquid plasma method	4.1	OCP: 0.85 V, Power density: 216 mW/cm^2^
[214]	Guo et al. (2005)	Pt/CB	Borohydride reduction method	3.8	Current density: 40 mA/cm^2^ at 0.45 V
[215]	Rao et al. (2011)	Pt/G, Pt_3_Co/G and Pt_3_Cr/G	Ethylene glycol reduction method	3.5, 4.2 and 4.3	Max. power density: 790, 875 and 985 mW/cm^2^
[216]	Yang et al. (2016)	Pt/CN-1 and Pt/CN-2	Hydrothermal synthesis	3, 40	ECSA: 60.9 and 25.7 m^2^/g MA: 313 and 132 mA/mg
[217]	Cho et al. (2012)	Pt/C and Pt_1_Ni_1_/C	Borohydride reduction method with acetate anions as stabilizer in anhydrous ethanol solvent	4, 2.7	ECSA: 24.4 and 28.2 m^2^/g Initial & final cell potentials: 0.69 & 0.45 V for Pt/C and 0.67 & 0.35 V for Pt_1_Ni_1_/C
[218]	Alegre et al. (2015)	Pt/CX, Pt/CB with formic acid	Impregnation method with two different reducing agents and microemulsion method	3.6, 4.6	ECSA: 38.6, 41.4 m^2^/g Peak mass activity: 367, 300 mA/mg
[219]	Lee et al. (2012)	Pt/C (M), Pt/C (P)	Modified polyol reduction method	1.3, 2.9	ECSA: 23, 16.4 m^2^/g Current density: 678 and 630 mA/cm^2^ at 0.6 V
[220]	Fu et al. (2015)	Pt-Co/MWCNTs	Ultrasonic enhanced synthesis	1.6	E_1/2_: 0.763 V
[221]	Hu et al. (2016)	Pt-Co nanoalloys	Tandem laser ablation synthesis in solution-galvanic replacement reaction (LASiS-GRR)	4.15 at pH 11	ECSA: 44.5 m^2^/g MA: 0.24 A/mg SA: 0.53 mA/cm^2^ E_1/2_: 0.875 V (vs. RHE)
[222]	Woo et al. (2011)	Pt-Co	Pulse electrodeposition (atomic ratio: 76:24)	3–5	Current density: 1.051 A/cm^2^ at 0.6 V
[223]	TrongchuanKij et al. (2011)	Pt-Co/C	Combined process of impregnation and seeding	2–3	Current & power density: 772 mA/cm^2^ & 460 mW/m^2^ at 0.6 V
[224]	He and Mukerjee (2010)	Pt-Co/C	Water-in-oil micro-emulsion	3–4	ECSA: 24 m^2^/g E_1/2_: 0.871 V MA: 1.242 A/µg SA: 5.175 mA/cm^2^ at 0.8 V
[225]	Liu et al. (2017)	Pt-Co/C-PANI	Microwave-assisted polyol method	3	E_1/2_: 0.943 V MA: 1.33 A/mg SA: 1.29 mA/cm^2^
[226]	Prasad et al. (2012)	Pt-MWCNT/PANI	Microwave synthesis	10–15	ECSA: 58.88 m^2^/g Current density: 1.7 mA/cm^2^
[227]	Chen et al. (2012)	Pt/C@PANI (20%), Pt/C@PANI (30%), Pt/C@PANI (50%)	*In situ* chemical oxidation polymerisation	2.5, 5, 14	ECSA: 67.5, 60.7, and 6.5 m^2^/g E_1/2_: 829, 819, and 761 mV MA: 68, 47 and 9 mA/mg at 0.85 V
[228]	Umezawa et al. (2017)	Pt_61_Ni_39_, Pt_47_Ni_53_ and Pt_20_Ni_80_	Plasma gas condensation cluster deposition (PGCCD) method	6–8	Power density: 100.1, 93.6, 65.7 mW/cm^2^
[229]	Wang et al. (2015)	Pt_3_-Ni/C, Pt_2_-Ni/C, Pt-Ni/C	Glycerol stabilized NaBH_4_ reduction at room temperature	2.4–3	ECSA: 72, 81, 45 m^2^/g MA: 0.192, 0.345, 0.083 mA/mg (at 0.512 V)
[230]	Do et al. (2015)	Pt_1_-Ni_1_/C	Electroless deposition method using NaBH_4_	4–8	ECSA: 18.06 m^2^/g
[231]	Lee et al. (2014)	PtNi/C(H), PtNi/C(A)	One-step sonochemical synthesis	3.7, 3.4	ECSA: 0.77, 0.63 cm^2^/g E_1/2_: 873, 847 mV
[232]	Rusnaeni et al. (2010)	PtNi/C	Polyol reduction method	5.71	ECSA: 36.56 cm^2^/mg SA: 99 µA/cm^2^
[233]	Kaewsai et al. (2018)	PtCr/C	Chemical reduction via seeding/ impregnation technique	3–10	Current density: 354 mA/cm^2^ at 0.6V Power density: 264 mW/cm^2^
[234]	Sahin et al. (2017)	PtCr/C	Modified microwave-assisted polyol method	3.43	Peak current density: 4.8, 5.1, 5.2 mA/cm^2^ at 690, 707 and 721 mV vs. RH at 20, 30 and 40 ^0^C, respectively
[235]	Min and Kim (2016)	Pt_1_Cr/C, Pt_3_Cr/C	An incipient wetness method	2–10	ECSA: 67 m^2^/g MA: 161, 203 A/g SA: 243, 308 µA/cm^2^
[236]	Taufany et al. (2011)	Pt_3_Cr_1_/C TD, Pt_3_Cr_1_/C EG	Combination of chemical reduction and thermal decomposition	3–3.5	ECSA: 43.44, 62.96 m^2^/g MA: 26.1, 6.99 A/g
[237]	Fedotov et al. (2013)	Pt/VXC-72	Magnetron-ion sputtering method	3.1	ECSA: 44 m^2^/g Power density: 550 mW/cm^2^ (at 0.55 V)
[238]	Bumaa et al. (2012)	Pt/PANI	Polyol method (EG)	4.36	Current density: 9.68 mA/cm^2^ at 0.36 V Maximum power density: 3.49 mW/cm^2^
[239]	Kim et al. (2018)	Pt/CP	Ultrasound irradiation (sonochemical) method	4.84	ECSA: 0.96 m^2^/gCurrent density: 0.413 A/cm^2^ at 0.6 V
[240]	Tegou et al. (2011)	PtNi/GC, PtCo/GC	Electrodeposition galvanic replacement method	7.29	ECSA: 3.6, 2.6 cm^2^/g
[241]	Hyun et al. (2013)	PtNi/C, PtCo/C, PtCu/C	Modified impregnation method	4.3, 5.8, 6.3	ECSA: 37.6, 37.5 and 24.0 m^2^/g; Max. power density: 0.587, 0.419, 0.448 W/cm^2^
[242]	Zhang et al. (2004)	Pt/C (HCHO)	Impregnation-reduction method	5.3	ECSA: 48.9 m^2^/g Maximum power density: 0.49 W/cm^2^ Current density: 906 mA/cm^2^

**Table 6:** Comparison of primary methods for the preparation of Pt-based catalysts.

Methods	Experimental parameters	Characteristics	
		Advantages	Disadvantages
Electro-Chemical	Electrochemical reduction by applying potentiostatic or galvanostatic excitation	Rapid reaction rate; Good loading control; Diverse morphology	Large particle size; Broad size distribution
Electroless deposition	Chemical reduction using a reducing agent or under H_2_ atmosphere	Facile and straightforward; Small particle size	Time-consuming; Impurity
PVD	Evaporation of target metal via plasma, electron or ion beam bombardment	Uniform size; Precise loading control; High utilization of Pt	Loose adhesion between Pt and carbon substrate; High cost of instrumentation
Irradiation-assisted	Synthesis under irradiation sources such as microwave, γ, UV and ultrasound	Uniform size; High dispersion; Pure and novel morphology	Need specific irradiation reactor

Further, the fabrication of nanocatalyst, dispersed over ultrasonically-prepared carbon material as support was obtained. Pollet described the functioning of ultrasound-based technology for the reduction. Sono-Tek Corporation (USA) found that the gas diffusion electrodes obtained by the ultrasonic-spray (US) method, especially at low Pt loadings in the range of 0.40–0.05 mg.cm^−2^, possessed enhanced properties when compared with those prepared commercially [210]. Further, Pollet confirmed ultrasound's effectiveness to produce PEM fuel cell electrodes containing (ultra)-low loading Pt [211]. Also, considering Pt/C catalyst, Pollet and Goh [212] studied the effects of ultrasound parameters (power, frequency, time) on the catalyst ink's electrochemical surface areas by comparing it with conventional mechanical stirring. They concluded that the optimised ultrasound parameters are quintessential to enhance the activity of catalytic ink. Even if ultrasound was used for a longer time, the composition and morphology were affected. Thus, utilising ultrasound for the fabrication of fuel cell is cost-effective, robust, environmentally friendly and time-saving. Hence, it is expected to be a promising solution compared to other conventional methods.

### 6.2 Refining of fuel

Although many alternative sources of power are under development, fossil fuel remains the largest and most widely used power source in many countries. Asphalts and sulphur and nitrogen-contaminated fossil fuels are found in large quantities worldwide, making them a persistent problem which needs to be solved. Where asphaltene was considered to be waste/harmful due to their property of being non-biodegradable, sulphur-contaminated fuel has been considered futile due to numerous reasons. It leads to ultimate failing of combustion engines, corrosion of vessels storing or transporting fuels and harming the catalysts used for fuels refining; besides the harmful emissions, it generates on its combustion.

The Clean Air Act 1964 caused the United States Environmental Protection Agency (US EPA) to limit the sulphur content of diesel fuel to 15 parts per million by weight (ppmw). For the refining industry, these standards cause challenges; for example, it becomes difficult to meet besides being expensive. Thus, to make economic profits, deasphalting, desulphurisation and hydrogenation utilizing intensified processes are standard practices. Many procedures have been previously suggested for such activities, namely, thermal scission, termination of radical reaction using particular chemicals, catalytic cracking, etc. Amongst these, catalytic cracking has been profoundly used. However, the problem with catalytic cracking is the requirement of high temperature and pressure, making the process techno-economically not viable. Application of ultrasound in this context has proved to solve this issue due to the favourable conditions it creates, some of which are:

• Implosion of cavitation microbubbles
• Rapid mixing
• Improved liquid–liquid interfacial area
• Generation of enormous vibrational energy in small volumes
• Weakened attractive forces, including hydrogen bonds, van der Waals, etc. that exist between larger molecules [243].

**Scheme 1:**
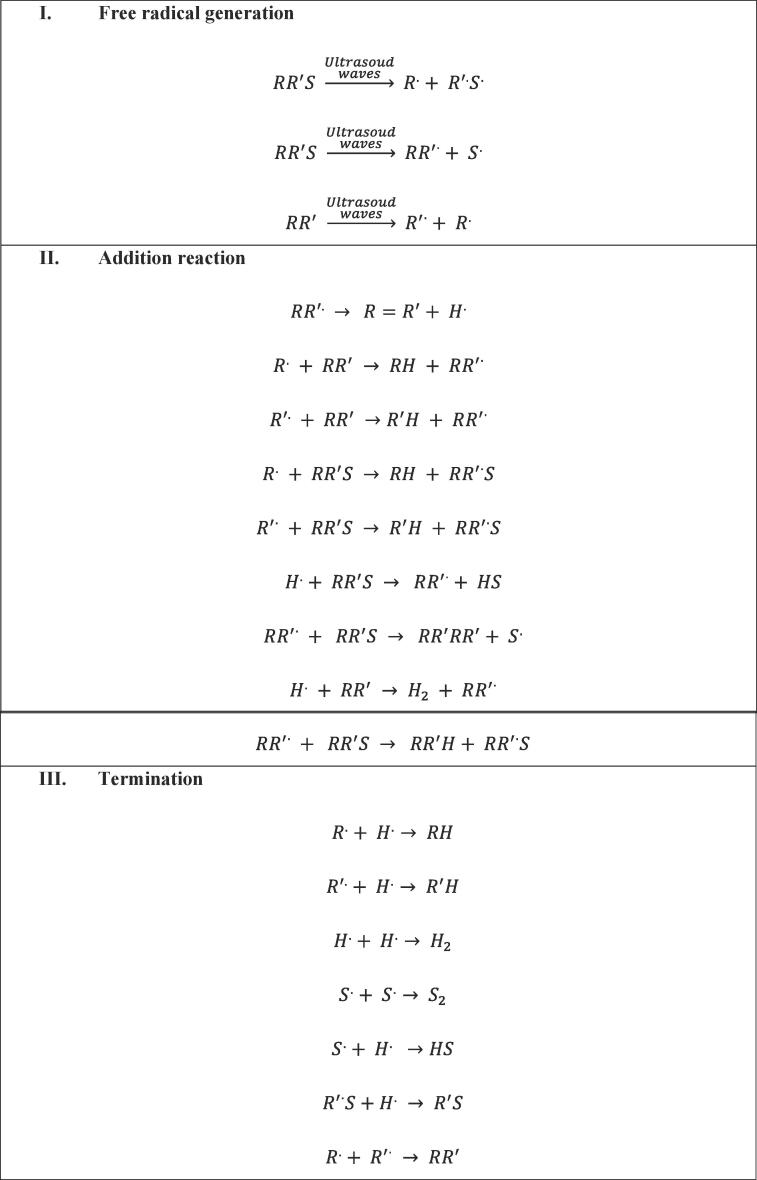
Mechanism of hydrodesulfurization.

These conditions lead to thermal scission of larger molecules such as asphaltene and hydrogenation of heavy crude, where the former is due to the produced hydrogen from hydrogen radicals. On the similar lines of upgrading hydrogenation of asphaltenes, hydrodesulfurization or oxidative desulphurisation is known to be the best approach for the desulphurisation of fossil fuels containing sulphur. In this approach, dibenzothiophene (DBT) and few sulphides are converted into sulphones which are comparatively more polar and can be removed via extraction or adsorption. If the organic sulphur present in the fuel is left out for combustion, they form SO_x_ and then cause harmful effects such as acid rain and devastation to flora and fauna. Thus, the organic sulphur is converted into gaseous hydrogen sulphide, which can then be oxidised to sulphur (solid) following the Claus process. After this processing, the remnants of H_2_S remain in the oil with its health hazards. The residual is mainly due to condensed multicyclic sulphur compounds, aromatic and other cyclic sulphur compounds that are not easily broken down. But with the assistance of ultrasound, even some of these compounds were observed to be broken because of the cavitation phenomenon, which causes the fused ring structure to open and unsaturation to decrease. Few researchers even attempted to combine two or more processes to eliminate sulphur in the fuel. Gunnerman in 2006, combined ultrasound and microwave and reported better results [244]. Scheme 1 illustrates the detailed mechanism of desulphurisation using ultrasound technology [245].

**Scheme 2:**
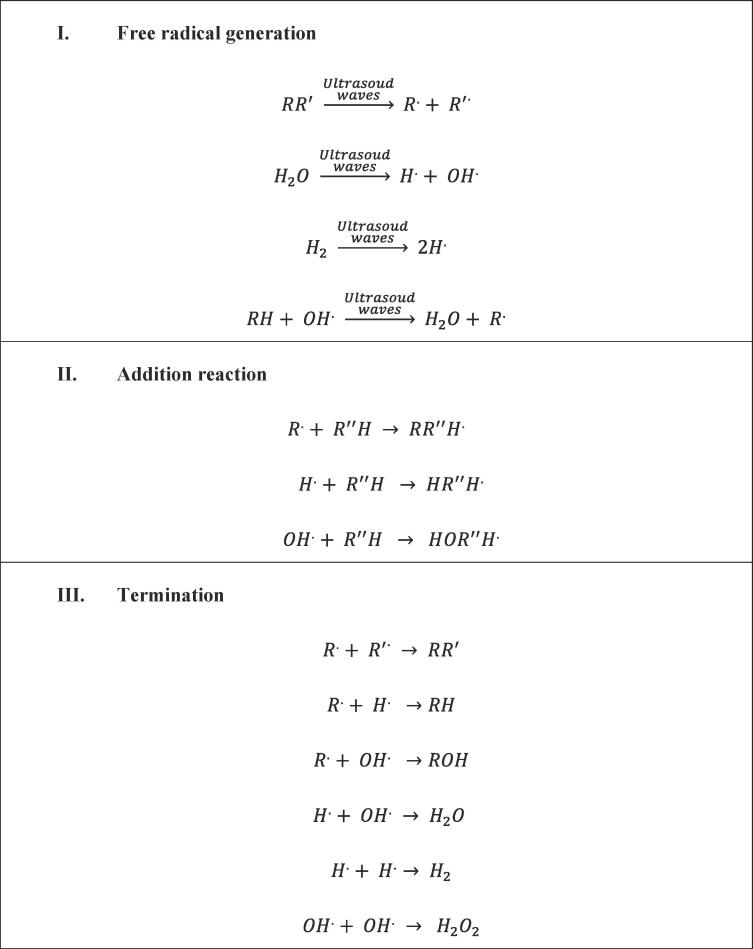
Mechanism for the transformation of asphaltene to gas oil and resins.

Lin et al. [196] described the mechanism behind the upgrading process of asphaltene (Scheme 2**).** When ultrasound is used, the only major requirements are hydrogen and hydroxyl radicals, which can be obtained from water, thereby making this technology robust and techno-economically viable. Due to the high selectivity of ultrasound-derived oxidation, the non-sulphur based components remain unchanged, which solves the issue of loss of important fuel components. Additionally, there are no requirements of surface-active agents. Similar to sulfur-bearing compounds, owing to the tendency of nitrogen-bearing compounds to harm the catalysts used for hydrocracking in refining crude oil, they are also removed from fossil fuels. This is achieved by hydrodenitrogenation, wherein due to the presence of metal sulphide catalysts, hydrogen-based treatment is performed [245], [246]. Apart from refining, ultrasound is found to aid in raising API (American Petroleum Institute) gravity, cetane index, lowering boiling point, destruction of compounds that are harmful to the fuel, and improving performance [246]. In Table 7**,** all the major research findings related to the application of ultrasound in refining crude oil have been reported. In some of the studies, ultrasound methods were compared with other conventional methods, whereas few involves coupling ultrasound with the conventional methods to improve the performance (Table 6). It is quite evident from all these studies that ultrasound is a promising solution to obtain cleaner fuels.

**Table 7:** Previous research works conducted in the area of application of ultrasound for the refining of fuels.

Year and Reference	Description of the methodology	Results
1993 [196]	Ultrasound, along with a surfactant and reducing agent for asphaltenes up-gradation.	For 15 min of sonication, the asphaltene content reduced by 35%.
2002 [197]	Ultrasound combined with H_2_O_2_ as an oxidizing agent, aqueous liquid (water) and catalysts (optional).	The efficiency increased from 60.5% to 99.2% depending on the conditions and the composition.
2003 [247]	Oxidative desulphurisation (OD) in comparison with ultrasound-assisted oxidative desulphurisation (UAOD).	OD + solvent extraction: Sulphur removal = 66.4% after 2.5 h OD + silica adsorption: Sulphur removal = 97.8% after 4 h UAOD + solvent extraction: Sulphur removal = 99.4% after 10 min.
2006 [245]	Ultrasound-assisted upgradation of heavy gas oil.	Optimised sonochemical conditions, without using solvent extraction at an amplitude of 50%, time = 10 min. Nitrogen conversion = 11% Sulphur conversion = 7% Viscosity reduction = 5%.
2007 [248]	Ultrasound-assisted hydrogenation of cyclohexene, biphenyl, and quinoline and hydrodesulfurization of benzothiophene.	Formic acid was used as a hydrogen precursor along with Pd/C catalyst at ambient temperature and pressure. Reduction in cyclohexane = 98% Reduction in biphenyl = 21% Reduction in quinoline = 19% Reduction in benzothiophene = 18%.
2007 [249]	Ultrasound method used for upgrading coal liquids, tar sands, oil shale, asphalt, and heavy oils.	Tar sands = 5% cumulative recovery Heavy oil - oil and resin fraction content increased from 68.3% up to 86.0% and reduced from 29.7% down to 15.6%. Coal liquids - oil and resin content increased up to 54.8%. Asphalt - oil and resin content increased up to 35%.
2008 [250]	Ultrasound coupled with Fenton’s reagent compared with conventional hydrotreating method of desulphurisation of diesel fuel.	Sulphur content dropped from 568.75 μg/g to 9.50 μg/g in 15 min.
2010 [251]	Ultrasound method used for desulphurisation of petroleum product feedstock and diesel oil and compared with conventional desulphurisation.	Using the conventional method: sulphur removal in petroleum product feedstock less than 82% and in diesel oil = 55% Using ultrasound method: sulphur removal = 99% in 9 min in petroleum product feedstock and > 75% in diesel oil.
2012 [252]	Power ultrasound coupled with activated carbon for desulphurisation of middle-distillate fuel.	Sulphur removal = 98% for jet propellant-8, 94% for diesel, and > 88% for ultralow-sulfur diesel.
2013 [252]	Optimum conditions for the ultrasound-assisted catalytic desulphurisation for gas oil.	90% reduction in 17 min at 62 ± 2 °C using 180.3 mmol of H_2_O_2_ and extraction by methanol.
2014 [253]	Effect of nitrogen in the ultrasound-assisted method for desulphurisation of diesel oil.	Sulphur removal = 69% and nitrogen removal = 84% after 9 min, using acetic acid and H_2_O_2_.
2015 [254]	Ultrasound method, along with methylate and water/fluoride mix for desulphurisation and metal removal.	Sulphur content reduced to below 5 ppm, and Hg, Pb, V, and Cd metals were removed.
2015 [255]	Ultrasound method along with FeCl_3_ catalyst and silica gel as an adsorbent for the desulphurisation of pyrolysis fuel obtained from tyres, plastics and lube oil.	Sulphur removal 87.5%. Without ultrasound, the removal was just 52.2%.
2016 [256]	Ultrasound method for denitrification of diesel oil using formic acid/hydrogen peroxide.	Nitrogen removal = 95.85% in 2 min.
2016 [257]	Ultrasound method for desulphurisation of DBT from model diesel oil.	Efficient removal of 98.35%.
2017 [258]	Ultrasound method for desulphurisation of DBT from model diesel oil.	95% removal in 80 sec.
2017 [259]	Ultrasound-assisted photocatalytic oxidative desulphurisation compared with mechanical agitation.	99.47% sulphur removal.
2019 [260]	Ultrasound coupled with Fenton's reagent, oxidative agent for desulphurisation of simulated fuel containing thiophene compared with the conventional method.	Removal was 96% during 80 min Conventional H_2_O_2_ removal = 22% Conventional Fenton removal = 40%.
2020 [261]	Ultrasound-assisted oxidative desulphurisation of heavy naptha using H_2_O_2_ and acetic acid.	89% removal from heavy naphtha containing 598.4 ppm of sulphur.

### 6.3 Biofuels

Due to the limited reserves of fossil fuels and is a significant cause of global warming, there is a need to develop benign and sustainable fuel/energy sources. Because of the ease with which chemical feedstock and liquid fuels can be produced from biomass and its availability in abundance, biomass is proved to fulfil this need. Biofuels are termed esters of simple alkyl fatty acids derived from biomass including waste vegetable oils and animal fat. Vegetable oils can’t be directly used as fuel because of their high viscosity, leading to an automobile engine's failure. Thus, conversion to biofuel is the requisite [262]. The usage of biodiesel commensurate an indirect use of solar energy [263]. Today, the two most familiar types of biofuels used from the first generation of biofuel technology are ethanol and biodiesel. The biofuel manufacturing is based upon the transesterification reaction, wherein triglyceride esters combine with alcohol such as methanol/ethanol in the presence or absence of catalysts (NaOH/KOH) to produce alkyl ester along with a by-product, glycerol. The alkyl esters are the required biofuels which are separated from glycerine using a centrifuge. Although biofuel has proved to be advantageous over fossil fuels due to their environmental friendliness, the process has been known to take a long time in the phase separation and esterification steps, causing biofuels to be expensive.

For scalability at the market level, biodiesel's high production cost is a major hurdle. Cutting the cost and increasing the rates of reaction could solve this problem. Mechanical stirring, a conventional method was utilized as a solution to this problem, and it resulted in success with the conversion of vegetable oils reaching 90–95% at times [264], but the drawbacks of this strategy are:

• Time-consuming
• Requires high pressures
• Requires reflux conditions

**Scheme 3:**
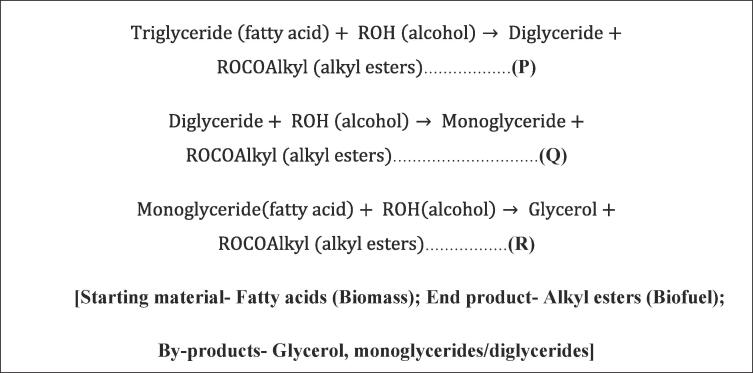
Mechanism of transesterification.

Low reaction rates and longer reaction time could be due to the immiscible mixture formed between the acid and the alcohol, causing mass transfer limitations. Cavitation phenomenon results in turbulence and liquid circulation, altering the enzyme's characteristics, reaction rates, and duration and mass transfer [265]. This is mainly caused because of the removal of lignin present in the biomass which binds cellulose and hemicelluloses together in plants. Once the lignin is removed, and the bond is broken, the mixing of the biomass and alcohol occurs faster, and thus the rates of reactions are enhanced [266]. Additionally, because of the collapse of cavitation bubbles, ultrasonic jets are created, causing the oil to mix with alcohol quickly [263]. Thus, ultrasound has been proved to be techno-economically effective for the intensified biodiesel synthesis, leading to economic benefits and reduced payback periods, provided that adequate and optimised conditions of temperatures and pressures are employed [267]. Stavarache et al. [263] studied the reaction mechanism of ultrasound-assisted transesterification of vegetable oils to produce biofuel (Scheme 3). The key finding is that when the ultrasound method is utilized, the rate-determining step (RDS) becomes step R rather than step Q, which is known to be the RDS for the mechanically activated route. This meant that diglycerides (DG’s) react quite fast to produce monoglycerides (MG’s) while MG’s react quite slowly. They suggested that this could be due to better affinity of MG’s for glycerol, causing them to stay closer to glycerol’s layer. Thus, MG’s become unable to contact the alcohol layer; thereby, the reaction rates are diminished.

Stavarache et al. [263] also concluded that sonication-assisted transesterification could be applicable for almost all vegetable oils. After this, numerous investigations focused on producing biofuels as accessible and as smooth as possible. Some of the notable research works are shown in Table 8. Also, Moholkar et al. [268] proposed a detailed physical and chemical mechanism for the ultrasound-assisted biofuel synthesis.

**Table 8:** Previous research works in the area of application of ultrasound in the production of biofuels.

Year and Reference	Description of the methodology		Results
2006 [269]	Biodiesel production via ultrasound-assisted extraction transesterification of disparate seed cakes.		No need of saponification Yield for cotton seeds = 85.5% Yield for sunflower seeds = 93% Yield for sesame seeds = 83.5%
2008 [270]	Conventional mechanical stirring compared with ultrasound-assisted in situ transesterification of sunflower seed oil for biodiesel production		Using ethanol, ester formation occurs. For ultrasound, 98% was obtained in 40 min and 88% in > 4 h using mechanical stirring
2008 [265]	Both hydrodynamic and acoustic cavitation were used for the generation of biodiesel from waste vegetable oil and were then compared with the conventional method		Cavitation proved to be more efficient than conventional and hydrodynamic cavitation proved to be superior. Using hydrodynamic cavitation resulted in 92% conversion in 90 min, and the conventional method took 69 h to obtain 90% conversion of oil.
2008 [271]	Sonochemical method compared with the conventional method for the methanolysis of Triolein		The reaction time required for the sonochemical method was 30 min, whereas the conventional method was 4 h.
2009 [272]	Ultrasound-assisted synthesis of ethyl esters from soyabean oil and ethanol		Maximum conversion of 91.8% was obtained using 30 min of ultrasound.
2010 [273]	Ultrasound-assisted biodiesel production using canola oil and methanol		The yield was>99% with 50 min of ultrasound.
2013 [274]	Ultrasound-assisted biodiesel production in batch and continuous mode compared with conventional stirring		Batch mode: For 90% yield, ultrasound was applied for 5 min, whereas stirring required 90 min. Continuous flow reactors in the presence of pulsed ultrasound: Reaction time = 18 sec for 90% conversion, and the rate was 300 times compared to the conventional method.
2014 [275]	Ultrasound-assisted synthesis of biofuel from biomass		Yield increased by 10–300% Reaction time reduced by 50–80% For algal biomass: Efficiency increased by 120–200%
2015 [276]	Bioethanol production from Parthenium hysterophorus biomass using ultrasound and conventional method		Conventional mechanical shaking: Ethanol production = 10.93 g/L, cell mass conc. 5.26 g/L in 18 h. Ultrasound-assisted synthesis: Ethanol production = 12.14 g/L, cell mass conc. 5.7 g/L in 10 h
2015 [277]	Bioethanol production from Norouzak seeds using the ultrasound method		Yield of 97.6% and the application in compression ignition engine was suggested.
2016 [278]	Ultrasound used for ethanol production following saccharification and fermentation of sugarcane bagasse		At 60% amplitude, ultrasound increased the enzyme's secretion, 90% ethanol yield while utilizing just 3 × 10^4^ J/g of energy.
2016 [279]	Ethanol produced from waste activated sludge using ultrasound		Waste sludge was ultrasonically treated for 30 min to recover essential nutrients. Hydrogen produced was 189.34 ml H_2_/g total sugar by E. Harbinense while ethanol recovered was 220.26 mg/g total sugar using those nutrients.
2017 [280]	Ultrasound used for ethanol production from sugarcane bagasse without enzymatic hydrolysis		Ultrasound + acid (H_2_SO_4_) treatment resulted in 820 mg/L of ethanol. Alkali (NaOH) treatment increased the yield to 911 mg/L.
2017 [281]	Ultrasound used for biodiesel production using microalgae oil		Without ultrasound, in 2 h, the yield was 63.9%; whereas using ultrasound, the yield increased to 97.6%.
2018 [282]	Ultrasound-assisted synthesis of triacetin via acetylation of glycerol		A selectivity of 100% was achieved. The SO_3_H-glycerol-carbon catalyst was reused for ten cycles.
2019 [283]	Ultrasound used for biodiesel production using solid food waste oil and compared with the results obtained using the conventional route		The yield of fatty acid methyl esters was 93.23% (w/w) Lesser reaction time (reduced by 40 min) Energy saving
2019 [284]	Ultrasound along with KI/ZnO catalyst was used for biodiesel production from mixed non-edible oils		Yield = 92.35 ± 1.08% The energy required for ultrasound method was 123.65 kJ/mol, and that for mechanical agitation was 135.4 kJ/mol

## 7 Conclusion

Sonochemistry is proved to be a robust, efficient and economical alternative in the field of nanoscience, due to the cavitation phenomenon, which is beneficial to obtain the desired particle size and surface area and enhance the physical and chemical properties of nanomaterials, better conversion rates, less time for the reaction, and lower energy consumption. These inherent advantages make them applicable in a range of technological fields in the future. This technique also seems to be economically available, making it superior to the already existing conventional methods. Commercialization of hydrogen energy is largely crippled due to not very efficient usage, production and storage techniques. To address the problems existing with hydrogen usage, fuel cells are the most beneficial, and widely recommended alternative for renewable energy. Usage of ultrasound and sonochemistry in numerous research works has shown improved energy output compared to other proposed methods.

Similarly, in hydrogen production, the use of ultrasound in processes such as ‘sono-hydrogen’, sonochemical and sonoelectrochemical methods have proved to be a greener alternative, where sonochemistry lies the heart of the solution. Both physical, chemical and electrochemical hydrogen storage techniques have benefited by applying sonochemical methods, demonstrating high surface area and high hydrogen storage capacity. Overall, from the studies that have already been reported the potential behind this technique could be realized. Thus the usage of sonochemical methods for hydrogen economy holds a bright future.

## 8 Future scope

Especially with the increasing technological advancements, the cost of sonication/ultrasound equipment has significantly reduced. As a result, extensive research has been carried out to apply sonochemistry to various synthesis methods. The researchers have also attempted to combine ultrasound with other technologies so that this hybrid technology's synergistic effect can lead to even better results. Microwave is another advanced technology with applications in diverse fields and could be useful in developing hybrid technologies. A research work elucidated combining ultrasound and microwave, which resulted in enhanced efficiencies, increased recoveries of valuable products etc. leading to biorefineries' advancements. It has also been suggested using ultrasound and microwave in a continuous plug flow reactor to use these intensified processes efficiently.

Application of sonochemistry in biomaterials, catalysis, drug delivery and nanomaterial synthesis are currently investigated to a greater extent. Some of the recent works include utilising ultrasound irradiation for biodiesel production using castor seeds, the ultrafast synthesis of WO_3_ nanoplates for water splitting, and the intensification of paracetamol synthesis from hydroquinone using ultrasound and thermal-sensitive droplets' synthesis for ultrasound imaging and drug delivery. Further, favourable results have been reported when ultrasound was utilised as an eco-friendly sustainable methodology for producing biohydrogen from dairy waste activated sludge.

Similarly, the application of ultrasound as a process intensification technique in various aspects of hydrogen energy and the economy is rising. It has been noted that such an application has already led to better results for hydrogen usage, production and storage. It is only a matter of time until researchers explore the usage of sonochemical methods for other previously not experimented systems. Adding to this, the phenomena of cavitation, which is the basis of sonochemical reactions have already been utilised through employing homogenisers towards applications such as pharmaceuticals, oil, wastewater treatment, food and beverages, among many others. Hence, it is expected that ultrasound due to its efficiency has enormous potential, and hence it will be extended to industry to obtain technologically viable solutions.

## 9 Authors statement

Authors' contributions

• Ujwal Kishor Zore, Sripadh Guptha Yedire, Narasimha Pandi, collected the literature and prepared the initial structure of the manuscript.
• Shirish Sonawane is the corresponding authors and contributed to improving the quality of the manuscript by reviewing and providing intellectual input.
• Sivakumar Manickam is the authors and contributed to improving the quality of the manuscript by reviewing and providing intellectual input.

## CRediT authorship contribution statement

**Ujwal Kishor Zore:** Hydrogen Energy data collection. **Sripadh Guptha Yedire:** Biofuel and fuel refinery data collection. **Narasimha Pandi:** Fuel cell data collection. **Sivakumar Manickam:** Reviewing and editing. **Shirish H. Sonawane:** Reviewing, editing and supervision.

## Declaration of Competing Interest

The authors declare that they have no known competing financial interests or personal relationships that could have appeared to influence the work reported in this paper.
